# DBDIpy: a Python library for processing of untargeted datasets from real-time plasma ionization mass spectrometry

**DOI:** 10.1093/bioinformatics/btad088

**Published:** 2023-02-14

**Authors:** Leopold Weidner, Daniel Hemmler, Michael Rychlik, Philippe Schmitt-Kopplin

**Affiliations:** Comprehensive Foodomics Platform, TUM School of Life Sciences, Technical University of Munich, Freising 85354, Germany; Analytical BioGeoChemistry, Helmholtz Zentrum Muenchen, Neuherberg 85764, Germany; Comprehensive Foodomics Platform, TUM School of Life Sciences, Technical University of Munich, Freising 85354, Germany; Analytical BioGeoChemistry, Helmholtz Zentrum Muenchen, Neuherberg 85764, Germany; Comprehensive Foodomics Platform, TUM School of Life Sciences, Technical University of Munich, Freising 85354, Germany; Comprehensive Foodomics Platform, TUM School of Life Sciences, Technical University of Munich, Freising 85354, Germany; Analytical BioGeoChemistry, Helmholtz Zentrum Muenchen, Neuherberg 85764, Germany

## Abstract

**Motivation:**

Plasma ionization is rapidly gaining popularity for mass spectrometry (MS)-based studies of volatiles and aerosols. However, data from plasma ionization are delicate to interpret as competing ionization pathways in the plasma create numerous ion species. There is no tool for detection of adducts and in-source fragments from plasma ionization data yet, which makes data evaluation ambiguous.

**Summary:**

We developed DBDIpy, a Python library for processing and formal analysis of untargeted, time-sensitive plasma ionization MS datasets. Its core functionality lies in the identification of in-source fragments and identification of rivaling ionization pathways of the same analytes in time-sensitive datasets. It further contains elementary functions for processing of untargeted metabolomics data and interfaces to an established ecosystem for analysis of MS data in Python.

**Availability and implementation:**

DBDIpy is implemented in Python (Version ≥ 3.7) and can be downloaded from PyPI the Python package repository (https://pypi.org/project/DBDIpy) or from GitHub (https://github.com/leopold-weidner/DBDIpy).

**Supplementary information:**

Supplementary data are available at *Bioinformatics* online.

## 1 Introduction

Application of plasma-based ion sources for real-time mass spectrometry (MS) shows exponential growth over the last years. This is because plasma ionization comes with relatively low instrumental cost, is simple to operate, very sensitive to a wide range of volatile analytes and is used in various fields like food processing-, environmental- or clinical breath research (Ayala-Cabrera *et al.*, 2022). Naturally, vendors of ion sources thrive to construct robust setups which reduce artifacts and maximize the generation of [M + H]^+^ pseudo-molecular ions in comparison to competing, undesirable adduct ions. However, the chemical composition of plasma is non-trivial (Adamovich *et al.*, 2017), highly dependent of surrounding atmospheric gases and the design of the ion source. These factors account for miscellaneous ionization and fragmentation pathways in plasma ion sources (Gyr *et al.*, 2019; Wolf *et al.*, 2016). Exemplarily, saturated analytes are prone to in-source oxygenation reactions or hydride abstractions. Some alternating ionization pathways still are even not understood up to date (Ayala-Cabrera *et al.*, 2022). Consecutively, the user is tempted to mistake multiple ion species of the same analytes as unrelated compounds, leading to ambiguous conclusions during the evaluation of untargeted datasets. In related analytical disciplines, the community developed tools to simplify complex data structures. Exemplarily, the CAMERA algorithm annotates adduct peaks in LC-MS data to form compound spectra for a better annotation of features (Kuhl *et al.*, 2012). Currently, there is no computational tool to process data from direct infusion plasma-based ionization available; even though the growing community is in need for harmonized data processing pipelines to handle the challenges of miscellaneous plasma ionization pathways. Therefore, we developed the open-source Python package DBDIpy. Inspired by data from dielectric barrier discharge ionization (DBDI), we provide a novel computational algorithm, to automate the interpretation and to reduce the size of datasets generated by plasma ionization. DBDIpy groups multiple ion species of the same analyte, removes spectral artifacts and facilitates the evaluation of convoluted data to the user.

## 2 Implementation

DBDIpy is designed to handle and process data obtained from untargeted high-resolution real-time plasma ionization MS. It has interfaces to the matchms-ecosystem; a popular Python package for processing of MS data (Huber *et al.*, 2020). Data loaded and preprocessed by matchms can be imported by DBDIpy for further analysis. The central algorithm of DBDIpy performs the grouping of systematically occurring ion species from the same.

### 2.1 Core functionality

The grouping of non-[M + H]^+^-ions with the pseudo-molecular ion is performed by a computational two-step open-search approach using the DBDIpy.identify_adducts() function: first, extracted ion chromatogram (XIC) shape similarities are calculated by computing pointwise Pearson correlation coefficient across all XIC pairs in the dataset. Second, highly correlated XICs are refined by mass difference analysis. Adducts and in-source fragments are identified from a set of pre-defined rules, which the user can flexibly customize. Exemplarily, the presence of two highly correlated XICs with a mass difference of 18.010565 ± the error of the mass spectrometer implicates an in-source water loss. The output of DBDIpy.identify_adducts() is a dictionary holding one data frame for each defined adduct type. The data frames contain information on the corresponding XICs matches such as correlation coefficient or mass difference. A comprehensive description of DBDIpy’s functions, the source code and an exemplary data-analysis workflow can be found on the GitHub repository and in the Supplementary Information. In brief, data are loaded and aligned by DBDIpy.import_spectra() via the matchms interface from matchms processing pipelines or from open file formats like .mgf. Followingly, missing values in the dataset are imputed by DBDIpy.impute_intensities() in preparation for adduct detection. This step consists of interpolating missing intensities within the signal region of the feature and of adding a noisy baseline to form uniform-length XICs. After this pre-treatment, adduct detection is performed as described above. The results of the adduct search can be visually inspected by calling DBDIpy.plot_adducts(): the temporal course of selected XICs, their correlation coefficients, mass differences and optionally supplied metadata will be shown to the user. This serves for validation of results and to investigate the grouped adduct systems. Finally, the DBDIpy.export_to_spectra() function permits data to be submitted to successive matchms data-handling or to be exported to open file formats.

### 2.2 Application

**Fig. 1.**
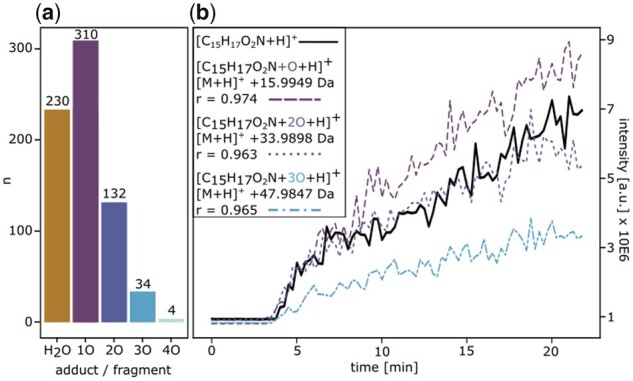
(**a**) Total number of in-source fragments and oxygen adducts detected by DBDIpy.identify_adducts() in an untargeted foodomics dataset of 4196 features (*r* > 0.95). (**b**) Temporal course of oxygen adducts generated during ionization of feature [C_15_H_17_O_2_N+H]^+^

To showcase the utility of DBDIpy, we performed a demonstrational data analysis. The demo data are from a foodomics study where wheat bread was roasted and thermal reaction products were monitored by DBDI-MS (Weidner *et al.*, 2023). It consists of 4196 features. After importing and preparing the data, DBDIpy.identify_adducts() was used to search for in-source water losses and for one to four oxygen adducts (correlation of *r* > 0.95). Figure 1a gives an overview of the quantity of identified adducts. In total, 710 potential adducts were identified, which corresponds to 17% of all features. This finding emphasizes the importance to perform adduct detection on untargeted plasma ionization datasets. Exemplarily, the temporal profile of an in-source oxidation series of one single compound annotated as [C_15_H_17_O_2_N+H]^+^ is shown in Figure 1b. An independent, network-based annotation workflow (Moritz *et al.*, 2017) was used to validate the finding. It confirmed the four mass signals to form a cluster of systematically oxidized and structurally related features.

## 3 Conclusion

We introduced DBDIpy as the first software tool for the identification and curation of in-source fragment ions and non-pseudomolecular ions from time-sensitive plasma ionization MS data. By uniting tied features to groups of adducts, DBDIpy reduces the data size of extensive untargeted datasets and facilitates their interpretation. Analytical chemists from various disciplines can integrate DBDIpy to their workflows as a key processing step to ameliorate their knowledge about convoluted spectra.

## Supplementary Material

btad088_Supplementary_Data

## Data Availability

DBDIpy is an open-source package and publicly available on GitHub https://github.com/leopold-weidner/DBDIpy. A demo dataset can be downloaded on Zendo under doi.org/10.5281/zenodo.7221088.

## References

[btad088-B1] Adamovich I. et al (2017) The 2017 plasma roadmap: low temperature plasma science and technology. J. Phys. D: Appl. Phys., 50, 323001.

[btad088-B2] Ayala-Cabrera J.F. et al (2022) Review on atmospheric pressure ionization sources for gas chromatography-mass spectrometry. Part I: Current ion source developments and improvements in ionization strategies. Anal. Chim. Acta, **1238**, 340353. 36464440 10.1016/j.aca.2022.340353

[btad088-B3] Gyr L. et al (2019) Characterization of a nitrogen-based dielectric barrier discharge ionization source for mass spectrometry reveals factors important for soft ionization. Anal. Chem., 91, 6865–6871. 31035763 10.1021/acs.analchem.9b01132

[btad088-B4] Huber F. et al (2020) matchms - processing and similarity evaluation of mass spectrometry data. J. Open Source Softw., 5, 2411.

[btad088-B5] Kuhl C. et al (2012) CAMERA: an integrated strategy for compound spectra extraction and annotation of liquid chromatography/mass spectrometry data sets. Anal. Chem., 84, 283–289. 22111785 10.1021/ac202450g PMC3658281

[btad088-B6] Moritz F. et al (2017) Characterization of poplar metabotypes via mass difference enrichment analysis. Plant Cell Environ., 40, 1057–1073. 27943315 10.1111/pce.12878

[btad088-B7] Weidner L. et al (2023) Real-time monitoring of miniaturized thermal food processing by advanced mass spectrometric techniques. Anal. Chem., 95, 1694–1702. 36602426 10.1021/acs.analchem.2c04874

[btad088-B8] Wolf J.C. et al (2016) A radical-mediated pathway for the formation of [M + H]+ in dielectric barrier discharge ionization. J. Am. Soc. Mass Spectrom., 27, 1468–1475. 27380388 10.1007/s13361-016-1420-2

