# Supplementary material for: DBDIpy: a Python library for processing of untargeted datasets from real-time plasma ionization mass spectrometry
Source: Bioinformatics. 2023 Feb 14;39(2):btad088. doi: 10.1093/bioinformatics/btad088 (PMC9942549; doi:10.1093/bioinformatics/btad088)
Supplement: btad088_Supplementary_Data [file btad088_supplementary_data.docx]

**DBDIpy: a Python library for processing of untargeted datasets from real-time dielectric barrier discharge ionization mass spectrometry**

Weidner Leopold, Hemmler Daniel, Rychlik Michael, Schmitt-Kopplin Philippe

**Supporting Information**

Table of content:

1 Acquisition of DBDI-MS data

2 Preprocessing of demonstrational data

3 Demonstrational DBDIpy workflow

4 Documentation and help files for DBDIpy’s functions

1 Acquisition of DBDI-MS data:

Approximately 2 mg of wheat bread dough (formulation: wheat flour, water, yeast, rapeseed oil, roasted barley malt, wheat malt, sea salt) from a local bakery were fermented at 34 °C, 80% humidity for 180 min. The samples were baked at 210 °C for 20 min in a laboratory-scale, analytical setup. The sample was fitted inside a borosilicate glass tube and introduced into a controllable GC-SPME-module directly connected to a SICRIT SC-30 ion source (both Plasmion GmbH, Augsburg, Germany). A flow of 1.0 L min^-1^ humidified Nitrogen and the negative pressure system of a 12 T solariX Fourier transformation ion cyclotron resonance mass spectrometer (FT-ICR-MS) (Bruker Daltonics, Bremen, Germany) actively transported thermal reaction products from the dough into the dielectric barrier discharge ion source. Ionization was performed at 1.5 kV and 15 kHz. For real-time data acquisition, the FT-ICR-MS was set to chromatographic mode. Data was acquisitioned between *m/z* 92 and 1000 at 4 megawords and with an ion accumulation time of 0.1 sec. In total, ten scans were accumulated per datapoint. Nitrogen at 2 L min^-1^ and 180 °C, was used as dry gas. A spray shield voltage of -400 V and a capillary voltage of 400 V were selected.

2 Preprocessing of demonstrational data:

Compass DataAnalysis 5.0 (Bruker Daltonics, Bremen, Germany) was used for internal calibration of raw data by reference mass lists. Singly charged ion signals with S/N > 6 were manually exported to mass lists and further pre-processed by an in-house workflow in R programming language (Version 4.2, R Foundation for Statistical Computing, Vienna, Austria) consisting of removal of side band artifacts, heavy isotope peaks and application of mass defect filtering. After alignment of mass lists to matrices holding mass spectrometric features, an in-house software tool was used to assign molecular formulae to the averaged *m/z*-values. Finally, 500 features were randomly selected from the matrix to form the demonstrational dataset for DBDIpy.

Demonstrational DBDIpy workflow:

In the following, we provide a comprehensive workflow to demonstrate the functions of DBDIpy (in markdown). It showcases all components from installation, the matchms interfaces and the core functionality.

## Tutorial

The following tutorial showcases an ordinary data analysis workflow by going through all functions of DBDIpy from loading data until visualization of correlation results. Therefore, we supplied a demo dataset which is publicly available [here](https://doi.org/10.5281/zenodo.7221089).

The demo data is from an experiments where wheat bread was roasted for 20 min and monitored by DBDI coupled to FT-ICR-MS. It consists of 500 randomly selected features.

![bitmap](https://user-images.githubusercontent.com/81673643/198022057-8b5da4b9-f6bd-43b7-9b6c-32fd119f93a7.png)

<p align = "center">

Fig.1 - Schematic DBDIpy workflow for in-source adduct and fragment detection: imported MS1 data are aligned, imputed and parsed to combined correlation and mass difference analysis.

</p>

### 1. Importing MS data

DBDIpy core functions utilize 2D tabular data. Raw mass spectra containing *m/z*-intensity-pairs first will need to be aligned to a DataFrame of features. We build features by using the ``align_spectra()`` function. ``align_spectra()`` is the interface to load data from open file formats such as .mgf, .mzML or .mzXML files via ``matchms.importing``.

If your data already is formatted accordingly, you can skip this step.

```python

##loading libraries for the tutorial

import os

import feather

import numpy as np

import pandas as pd

import DBDIpy as dbdi

from matchms.importing import load_from_mgf

from matchms.exporting import save_as_mgf

##importing the downloaded .mgf files from demo data by matchms

demo_path = "" #enter path to demo dataset

demo_mgf = os.path.join(demo_path, "example_dataset.mgf")

spectrums = list(load_from_mgf(demo_mgf))

##align the listed Spectra

specs_aligned = dbdi.align_spectra(spec = spectrums, ppm_window = 2)

```

We first imported the demo MS1 data into a list of ``matchms.Spectra`` objects. At this place you can run your personal ``matchms`` preprocessing pipelines or manually apply filters like noise reduction.

By aplication of ``align_spectra()``, we transformed the list of spectra objects to a two-dimensional ``pandas.DataFrame``. Now you have a column for each mass spectrometric scan and features are aligned to rows. The first column shows the mean *m/z* of a feature.

If a signal was not detected in a scan, the according field will be set to an instance of ``np.nan``.

Remember to set the ``ppm_window`` parameter according to the resolution of you mass spectrometric system.

We now can inspect the aligned data, e.g. by running:

```python

specs_aligned.describe()

specs_aligned.info()

```

Several metabolomics data processing steps can be applied here if not already performed in ``matchms``. These might include application of noise-cutoffs, feature selection based on missing values, normalization or many others.

``specs_aligned.isnull().values.any()`` will give us an idea if there are missing values in the data. These cannot be handled by successive DBDIpy functions and most machine learning algorithms, so we need to impute them.

### 2. Imputation of missing values

``impute_intensities()`` will assure that after imputation we will have a set of uniform length extracted ion chromatograms (XIC) in our DataFrame. This is an important prerequisite for pointwise correlation calculation and for many tools handling time series data.

Missing values in our feature table will be imputed by a two-stage imputation algorithm.

- First, missing values within the detected signal region are interpolated in between.

- Second, a noisy baseline is generated for all XIC to be of uniform length which the length of the longest XIC in the dataset.

The function lets the user decide which imputation method to use. Default mode is ``linear``, however several others are available.

```python

feature_mz = specs_aligned["mean"]

specs_aligned = specs_aligned.drop("mean", axis = 1)

##impute the dataset

specs_imputed = dbdi.impute_intensities(df = specs_aligned, method = "linear")

```

Now ``specs_imputed`` does not contain any missing values anymore and is ready for adduct and in-source fragment detection.

```python

##check if NaN are present in DataFrame

specs_imputed.isnull().values.any()

Out[]: False

```

### 3. Detection of adducts and in-source fragments

Based on the ``specs_imputed``, we compute pointwise correlation of XIC traces to identify in-source adducts or in-source fragments generated during the DBD ionization process. The identification is performed in a two-step procedure:

- First, calculation of pointwise intensity correlation identifies feature groups with matching temporal intensity profiles through the experiment.

- Second, (exact) mass differences are used to refine the nature of potential candidates.

By default, ``identify_adducts()`` searches for [M-H<sub>2</sub>O+H]<sup>+</sup>, [M+O<sub>1</sub>+H]<sup>+</sup> and [M+O<sub>2</sub>+H]<sup>+</sup>.

For demonstrational purposes we also want to search for [M+O<sub>3</sub>+H]<sup>+</sup> in this example.

Note that ``identify_adducts()`` has a variety of other parameters which allow high user customization. See the help file of the functions for details.

```python

##prepare a DataFrame to search for O3-adducts

adduct_rule = pd.DataFrame({'deltamz': [47.984744],'motive': ["O3"]})

##identify in-source fragments and adducts

search_res = dbdi.identify_adducts(df = specs_imputed, masses = feature_mz, custom_adducts = adduct_rule,

method = "pearson", threshold = 0.9, mass_error = 2)

```

The function will return a dictionary holding one DataFrame for each adduct type that was defined. A typical output looks like the following:

```python

##output search results

search_res

Out[24]:

{'O': base_mz base_index match_mz match_index mzdiff corr

19 215.11789 24 231.11280 ID40 15.99491 0.963228

310 224.10699 33 240.10191 ID51 15.99492 0.939139

605 231.11280 39 215.11789 ID25 15.99491 0.963228

1413 240.10191 50 224.10699 ID34 15.99492 0.939139

1668 244.13321 55 260.12812 ID67 15.99491 0.976541,

...

'O2': base_mz base_index match_mz match_index mzdiff corr

1437 240.10191 50 272.09174 ID77 31.98983 0.988866

1677 244.13321 55 276.12304 ID84 31.98983 0.972251

2362 260.12812 66 292.11795 ID100 31.98983 0.964096

3024 272.09174 76 240.10191 ID51 31.98983 0.988866

3354 276.12304 83 244.13321 ID56 31.98983 0.972251,

...

'H2O': base_mz base_index match_mz match_index mzdiff corr

621 231.11280 39 249.12337 ID60 18.01057 0.933640

1883 249.12337 59 231.11280 ID40 18.01057 0.933640

3263 275.13902 82 293.14958 ID102 18.01056 0.948774

4775 293.14958 101 275.13902 ID83 18.01056 0.948774

5573 300.08665 112 318.09722 ID140 18.01057 0.905907

...

'O3': base_mz base_index match_mz match_index mzdiff corr

320 224.10699 33 272.09174 ID77 47.98475 0.924362

1688 244.13321 55 292.11795 ID100 47.98474 0.964896

3013 272.09174 76 224.10699 ID34 47.98475 0.924362

4631 292.11795 99 244.13321 ID56 47.98474 0.964896

13597 438.28502 308 486.26976 ID356 47.98474 0.935359

...

````

The ``base_mz`` and ``base_index`` column give us the index of the features which correlates with a correlation partner specified in ``match_mz`` and ``match_index``.

The mass difference between both is given for validation purpose and the correlation coefficient between both features is listed.

Now we can for example search series of Oxygen adducts of a single analyte:

```python

##search for oxygenation series

two_adducts = np.intersect1d(search_res["O"]["base_index"], np.intersect1d(search_res["O"]["base_index"],search_res["O2"]["base_index"]))

three_adducts = np.intersect1d(two_adducts , search_res["O3"]["base_index"])

three_adducts

Out[33]: array([55, 99], dtype=int64)

```

This tells us that features 55 and 99 both putatively have [M+O<sub>1-3</sub>+H]<sup>+</sup> adduct ions with correlations of R<sup>2</sup> > 0.9 in our dataset.

Let's visualize this finding!

### 4. Visualization of correlation results

Now that we putatively identified some related ions of a single analyte, we want to check their temporal response during the baking experiment.

Therefore, we can use the ``plot_adducts()`` function to conveniently draw XICs.

The demo dataset even comes along with some annotated metadata for our features, so we can decorate the plot and check our previous results!

```python

##load annotation metadta

demo_path = "" #enter path to demo dataset

demo_meta = os.path.join(demo_path, "example_metadata.feather")

annotation_metadata = feather.read_dataframe(demo_meta)

##plot the XIC

dbdi.plot_adducts(IDs = [55,66,83,99], df = specs_imputed, metadata = annotation_metadata, transform = True)

```

<p align="center">

<img width="600" height="288" src="https://user-images.githubusercontent.com/81673643/198047792-9a9019ab-5c00-4365-a25c-2cbcd0d3d20f.png">

</p>

<p align = "center">

Fig.2 - XIC plots for features 55, 66, 83 and 99 which have highly correlated intensity profile through the baking experiment.

</p>

We see that the XIC traces show a similar intensity profile through the experiment. The plot further tells us the correlation coefficients of the identified adducts.

From the metadata we can see that the detected mass signals were previously annotated as C<sub>15</sub>H<sub>17</sub>O<sub>2-5</sub>N which tells us that we most probably found an Oxgen-adduct series.

If MS2 data was recorded during the experiment we now can go on further and compare fragment spectra to reassure the identifications. You might find [ms2deepscore](https://github.com/matchms/ms2deepscore) to be a usefull library to do so in an automated way.

### 5. Exporting tabular MS data to match.Spectra objects

If you want to export your (imputed) tabular data to ``matchms.Spectra`` objects, you can do so by calling the ``export_to_spectra()`` function. We just need to re-add a column containing *m/z* values of the features.

This gives you access to the matchms suite and enables you to safe your mass spectrometric data to open file formats.

Hint: you can manually add some metadata after construction of the list of spectra.

```python

##export tabular MS data back to list of spectrums.

specs_imputed["mean"] = feature_mz

speclist = dbdi.export_to_spectra(df = specs_imputed, mzcol = 88)

##write processed data to .mgf file

save_as_mgf(speclist, "DBDIpy_processed_spectra.mgf")

```

We hope you liked this quick introduction into DBDIpy and will find its functions helpful and inspiring on your way to work through data from direct infusion mass spectrometry. Of course, the functions are applicable to all sort of ionisation mechanisms and you can modify the set of adducts to search in accordance to your source.

If you have open questions left about functions, their parameter or the algorithms we invite you to read through the built-in help files. If this does not clarify the issues, please do not hesitate to get in touch with us!

4 Documentation and help files for DBDIpy’s functions:

Here we provide an extensive description of DBDIpy’s functions including their parameters, default values and output objects.

align_spectra(spec, ppm_window = 2):

"""Feature building from a list of spectra.

Aligns detected peaks in a list of matchms.Spectra objects into two-dimensional tabular data.

Utilizes data loaded by matchms.importing module and connects to matchMS (pre-)processing workflows.

Parameters

----------

spec : list of matchms.Spectra

Spectra imported by matchms from mass spectrometric experiments

(MS1) of instance matchms.Spectrum.

ppm_window : float, optional

Window for mass alignment in ppm. Default is 2 ppm.

Returns

-------

A two-dimensional pd.DataFrames containing aligned mass spectrometric features.

The first column contains mean m/z values of peaks across all scans followed by

column-wise arranged signal intensities.

Absence of a signal in a scan results in filling the table with nan instead.

See Also

--------

matchms.importing : For information about reading mass spectrometric data into Python.

"""

export_to_spectra(df, mzcol = 0):

"""Builds a list of spectra from tabular data.

Splits a two-dimensional pd.DataFrames containing aligned mass spectrometric features

to a list of matchms.Spectrum objects for further processing or exporting to .mgf-files.

Parameters

----------

df : pd.DataFrame

A DataFrame containing tabular mass spectrometric data.

The first column (by default) contains mass to charge ratios,

successive columns contain corresponting signal intensities

of each mass spectrometric scan.

mzcol : int, optional

Position of the column containing m/z information of the features.

Default is 0.

Returns

-------

A a list containing a matchms.Spectrum object for each column of the input

DataFrame except for the m/z column.

See Also

--------

matchms.exporting : For information about writing mass spectrometric data

to open file formats.

"""

identify_adducts(df, masses, custom_adducts = None, method = "pearson", threshold = 0.90, mass_error = 2):

"""Finds different ion species of a single analyte molecule.

Computes pointwise correlation of XIC traces to identify in-source adducts or in-source fragments

generated during direct infusion mass spectrometric data aquisition.

Putative identification of adducts and fragments is based on a correlation threshold and mass differences.

Parameters

----------

df : pd.DataFrame

A DataFrame of equal-length ion traces formated as rows.

Input DataFrame can be provided by align_spectra() and impute_intensities().

masses : pd.series

A series of m/z values for specification of adduct or fragment types.

Use of theoretic masses is strongly recommended for higher precission.

If theoretic masses are not available, adapt mass_error

in accordance to the type of your mass analyzer.

adduct_rules : pd.DataFrame, optional

The function searches for 1-Oxygen and 2-Oxygen adducts

and in-source water-losses by default.

Custom adduct rules need to be specified in a DataFrame as following:

custom_adducts = pd.DataFrame({'deltamz': [mz1, mz2, mz3],

'motive': ["motive1", "motive2", "motive3"]}).

method : str, {'pearson’, ‘spearman’, ‘kendall’}

Correlation method for pointwise compariation of XIC traces.

threshold : float

Correlation treshold to associate two ions. Default is 0.9.

mass_error : int or float

Tolerance of the mass spectrometer in ppm. Default is 2 ppm.

Returns

-------

A dictionary of DataFrames containing pairwise information about correlation of XIC traces and putatively identified in-source adducts or in-source fragments.

Raises

------

ValueError

If the input DataFrame contains missing values.

See Also

--------

align_spectra(), impute_intensities() : For preparation of the input data.

"""

impute_intensities(df, method = "linear"):

"""Fills nan values in data tables.

Imputes NaN values contained in a DataFrame consisting of aligned mass spectra.

Input DataFrame can be provided by align_spectra().

Extracted Ion Chromatograms often are not of the same length. To generate a set of

uniform-length ion intensity series, a multi-step imputation approach is used:

I) Missing values within the detected signal region are interpolated.

II) A noisy baseline is added for all XIC to be of uniform length which

the length of the longest XIC in the dataset.

The returned DataFrame is suitable for time series analysis or other multivariate statistics.

Parameters

----------

df : pd.DataFrame

A DataFrame containing missing (NaN) values to be imputed.

Input DataFrame can be provided by align_spectra().

method : str

Intepolation method to be used; default is "linear".

Supported imputation methods are all methods from

pandas.DataFrame.interpolate() and

scipy.interpolate.interp1d().

Returns

-------

A DataFrame of equal length ion intensity series without NaN.

See Also

--------

align_spectra() : For preparation of the input data.

"""

plot_adducts(IDs, df, metadata = None, transform = False):

"""Visualizes identified adducts or in-source fragments.

A graphical tool for the visualization of correlated XIC traces identified by

identify_adducts().

The temporal evolution of selected features is plotted to inspect correlation results

and adduct information.

Parameters

----------

IDs : list

A list of IDs (indices) to select correlated features from df.

df: pd.DataFrame

A two-dimensional DataFrame containing aligned mass spectrometric features

e.g. generated by align_spectra().

metadata : pd.DataFrame, optional

A DataFrame containing annotated metadata for the intensity Data.Frame.

Should contain a column called "mol_formula" for annotation of the plot.

transform : bool, optional

Whether plotted intensities should be scaled by a log2 function.

Returns

-------

Shows a 2D-scatterplot of XIC and returns it as a matplotlib.figure object for

individual modification.

See Also

--------

align_spectra() : For preparation of the input data.

identify_aducts() : For identification of multiple ion species from one compound.

matplotlib.plt : For further customization of the returned plot object

"""
